# Effect of Thermal Aging on Color Stability and Mechanical Properties of High-Density CAD/CAM Polymers Utilized for Provisional Restorations

**DOI:** 10.3390/jfb16060223

**Published:** 2025-06-15

**Authors:** Rasha Alharthi, Ali Robaian Alqahtani, Abdullah Mohammed Alshehri, Abdulrahman Almalki, Heba Wageh Abozaed, Eman Mohamed Raffat Hussein, Tarek Ahmed Soliman

**Affiliations:** 1 Clinical Dental Science Department, College of Dentistry, Princess Nourah bint Abdulrahman University, Riyadh 11671, Saudi Arabia; rsalharthi@pnu.edu.sa; 2 Conservative Dental Science Department, College of Dentistry, Prince Sattam bin Abdulaziz University, Al-Kharj 11942, Saudi Arabia; ali.alqahtani@psau.edu.sa (A.R.A.);; 3 Prosthetic Dental Department, College of Dentistry, Prince Sattam bin Abdulaziz University, Al-Kharj 11942, Saudi Arabia; 4 Prosthetic Dental Department, Faculty of Dentistry, National Benha University, Benha 13511, Egypt; emanraffat70@gmail.com

**Keywords:** aging, color stability, flexural properties, provisional restoration

## Abstract

Background: This study evaluated and compared the effects of thermal aging on the color stability and mechanical properties of CAD/CAM polymers utilized for provisional restorations. Material and Methods: Three CAD/CAM polymers in this study: CAD-Temp (CAT), Everest C-Temp (CT), and PEEK (PK). Forty specimens of each material were randomly assigned to two subgroups. Subgroup A was immersed in distilled water for 24 h, whereas Subgroup B was subjected to 5000 thermal cycles. The color stability, flexural strength (FS), survival probability, and microstructures were evaluated following thermal cycling. Data analysis was conducted utilizing two-way ANOVA along with Tukey’s test. Results: The CAT (3.74 ± 0.39) and CT (3.51 ± 0.54) groups exhibited the highest color variations, while PEEK (2.95 ± 0.45) showed the lowest color change. The baseline groups showed that the CT group had the highest flexural strength value (*p* < 0.05). The flexural strength values of CAT and CT groups significantly decreased (*p* < 0.05) following thermal cycling. No significant decrease in FS was observed following thermal cycling in the Pk group (*p* = 0.16). Conclusions: The color measurement and flexural strength outcomes were significantly influenced by CAD/CAM materials and thermal cycling. The CT group demonstrated superior flexural strength compared to the other groups, both before and after thermal cycling. The PK group shows the lowest color change compared to other groups. Regardless of aging condition, C-Temp and PEEK materials recorded the highest survival probability, a 95% significance level compared to CAD-Temp.

## 1. Introduction

Provisional restorations are commonly utilized in the treatment of fixed partial dentures. Prior to the final dental prosthesis placement, they aid in the maintenance of occlusal stability and the evaluation of treatment effectiveness in terms of aesthetic, functional, and therapeutic benefits [1]. Provisional restorations are produced using either traditional methods or CAD/CAM technology. PMMA is the most widely used polymer. However, heat generation during polymerization can lead to pulp damage and shrinkage. Consequently, bis-acryl resin composites have surpassed PMMA. Fabrication of provisional restorations using CAD/CAM polymers has recently gained popularity due to its increased precision and decreased time requirements [2,3]. High-density CAD/CAM polymers are long-term provisional restorations produced from cross-linked CAD/CAM polymer materials. These materials are polymerized under controlled and standardized conditions, utilizing optimized pressure and temperature parameters, which enhance their properties beyond those achieved through conventional polymerization [4].

Long-term provisional restorations play a crucial role in various clinical scenarios, including implant-assisted prostheses and occlusal rehabilitation, where they are subjected to considerable functional loads [5,6,7]. Clinicians should consider material type, mechanical properties, and oral environmental variables when selecting long-term provisional restorative materials [8,9]. Provisional restorations play a crucial role in dental rehabilitation cases. They must have appropriate mechanical properties that enable them to resist repeated functional forces in the oral environment. Therefore, understanding their mechanical properties is critical for anticipating their behavior and thus their clinical performance in an oral setting [8,9]. The color appearance is particularly crucial for restorations intended for long-term use [10,11,12,13]. Recent advancements in manufacturing technologies, particularly high-density polymers, present significant opportunities for oral rehabilitation. The CAD/CAM approach is used to fabricate long-term provisional restorations using a crosslinked PMMA block. Additionally, this material is utilized for applications ranging from single-tooth restorations to four-unit fixed dental prostheses and implant-supported restorations [8,9,10].

Although CAD/CAM provisional restorations demonstrate significant promise, their durability in the oral environment could be influenced. Provisional restorations frequently encounter oral conditions, such as temperature fluctuations and repetitive occlusal forces. Thermal and occlusal stresses may deteriorate the surfaces of materials and undermine their mechanical properties when hot and cold beverages are ingested in the oral cavity [14,15,16,17]. Consideration of color change is essential to guarantee the lasting aesthetic success of a provisional restoration. Besides color change, flexural properties significantly influence the longevity of these restorations, as a higher flexural strength indicates a greater capacity to withstand increased forces [16,18]. The flexural properties and color stability of provisional restorations are crucial, particularly in cases involving patients with parafunctional habits or requiring long-term use [18,19]. Although CAD/CAM provisional restorations appear to be highly promising, their long-term survival rate in the oral environment might be compromised due to their diverse micro-structures. CAD/CAM materials, including acrylate polymer, fiberglass-reinforced polymer, and polyether ether ketone, have been introduced to solve this issue, with claims of improved mechanical properties and durability [18,19,20,21]. However, there is little information on their physico-mechanical properties. Consequently, our study examined the influence of thermocycling on the color stability and flexural properties of three different CAD/CAM polymers used for long-term provisional restorative materials. The null hypotheses examined were that thermocycling does not have a significant impact on (1) the color stability and (2) the flexural properties of three different CAD/CAM materials utilized for long-term interim restorations.

## 2. Materials and Methods

The different CAD/CAM polymers used in this study are listed in Table 1. These include CAD-Temp (polyacrylate polymer; CAT), Everest C-Temp (fiberglass-reinforced polymer; CT), and BioHPP (polyether ether ketone; PEEK; PK).

### 2.1. Specimen’s Preparation and Color Measurement

Forty specimens of each CAD/CAM material, measuring 15 mm × 15 mm × 1 mm were produced by sectioning the CAD/CAM blocks using a precision low-speed saw (Techcut4, Remscheid, Germany). Specimens’ finishing and polishing were performed according to the manufacturer’s instructions. Specimens were finished using silicon carbide papers of varying grit sizes (600–2000 grits) while being cooled with water, followed by a 3-min ultrasonic washing in deionized water to achieve a uniform standard [8]. Finally, they were polished with diamond polishing paste (Grinder polisher Metaserve 250; BUEHLER, Lake Bluff, IL, USA) at a constant speed of 8000 rpm for 60 s to achieve a uniform standard [8,9]. The specimen dimensions were confirmed to be accurate with a digital caliper and subsequently distributed randomly into two subgroups. Subgroup A was immersed in distilled water for 24 h, whereas Subgroup B was subjected to 5000 thermal cycles ranging from 5 to 55 °C for a duration of 30 s (SD Mechatronic GmbH, Feldkirchen Westerham, Bayern, Germany).

A spectrophotometer (5000 UV-Vis-NIR, Agilent Technologies, Santa Clara, CA, USA) equipped with an illuminant D65 and a spectral range of 175–3300 nm was employed to assess color on a white background. The illumination settings were in accordance with CIE diffuse/8° geometry, and the aperture diameter was 3 mm. To mitigate the edge-loss effect, a concentrated sucrose solution was employed to improve the optical connection between the sample and the background. Calibration was conducted prior to each color measurement. The specimens’ color was assessed utilizing the L*a*b* color system. In this approach, L* denotes lightness, ranging from white to black. The symbol a* represents the red-green spectrum, with positive values indicating red and negative values indicating green. The b* denotes the yellow-blue spectrum, where positive values signify yellow and negative values represent blue.

For each sample, three measurements were taken, and the average values of L*, a*, and b* were determined. The color difference (ΔE) between specimens before and after thermocycling was calculated using the CIELAB equation [14,15]: ${\Delta E*=[{\left(\Delta L\right)}^{2}+{\left(\Delta a\right)}^{2}+{\left(\Delta b\right)}^{2}]}^{1/2}$ where L* = lightness (0–100), a* = change the color of the axis (red/green), and b* = color variation axis (yellow/blue). The degree of color change was quantified using the National Bureau of Standards (NBS) approach (Table 2) [22]. The color change was compared to a clinical standard by converting the ΔE* values into NBS units: NBS = ΔE × 0.9 [23,24].

### 2.2. Mechanical Properties: Flexural Properties and Weibull Analysis

**Figure 1:**
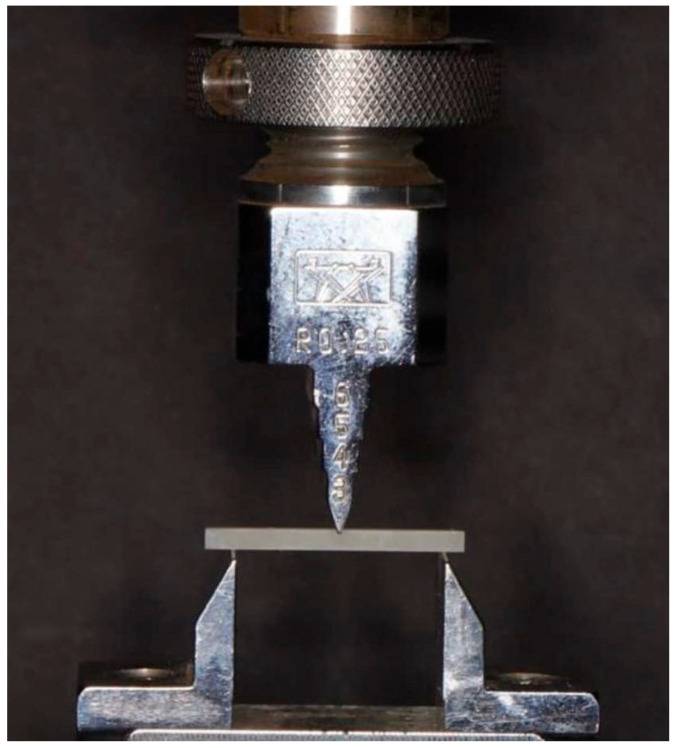
Flexural strength testing.

Twenty bar-shaped specimens were fabricated from each material, with dimensions of 25 mm × 2 mm × 2 mm^3^ [25]. The Instron universal testing machine was utilized to conduct flexural strength testing (Figure 1). The specimens were firmly positioned in a holding device featuring a support span of 20 mm. A compressive force was applied perpendicularly to the central regions of the specimens at a crosshead speed of 1.0 mm/min. Flexural strength (MPa) measurements were calculated according to the following equation:

Flexural strength $(\sigma_{s})=\frac{3PL}{{2BD}^{2}}$ where P (N) is the maximum load at fracture, L is the span between the two supporting ends, B is the width of the bar, and D is the height of the bar, and d is the deflection corresponding to load P [26,27]. The flexural strength data were analyzed through Weibull analysis to determine the flexural strength at various survival probabilities (P_s_). The formula for the Weibull distribution can be expressed as P_s_ = EXP [−(σ/σ_0_) m]. In this equation, P_s_ indicates the survival probability at a specific shear stress, σ represents the FS under any stress, σ_0_ refers to the characteristic flexural strength, and m is the Weibull modulus [28].

### 2.3. Scanning Electron Microscopy Analysis

Three samples of each material measuring 10 mm × 10 mm × 1 mm were produced. They were cleaned in an ultrasonic bath containing 96% ethanol for two minutes and subsequently air-dried. The samples were analyzed using a scanning electron microscope (Jeol-JSM-6510, Tokyo, Japan) at a magnification of 500× after being attached to metallic stubs and coated with gold using a sputter coater for qualitative assessment [8].

### 2.4. Statistical Analysis

The assumptions of normality and homogeneity of variance were confirmed by Shapiro–Wilk and Levene’s tests. Statistical analyses were conducted using SPSS 22.0 (IBM statistics). Color measurements were evaluated using one-way ANOVA. To identify statistically significant differences, flexural strength was assessed using two-way ANOVA. Post-hoc analyses were conducted using Tukey’s significant difference test. A significant level of 5% was set for all statistical tests. Additionally, the flexural strength data was utilized in a Weibull analysis to determine the Weibull modulus (m) and characteristic flexural strength (σ_0_).

## 3. Results

### 3.1. Color Measurements

Color changes (ΔE*) for all CAD/CAM materials are shown in Table 3. “To compare color change to clinical standards, ΔE values were transformed to NBS units (Table 2): NBS = ΔE × 0.9” [20,21,22]. According to the NBS system, the higher color changes were recorded for CAT (3.74 ± 0.39) and CT (3.51 ± 0.54) groups. Both CAT and CT groups showed color change above the “marked change scale” (3.0–6.0) due to the thermocycling. However, the PK group revealed color change (2.95 ± 0.45) in the “perceivable scale” (1.5–3.0). The greatest color change was recorded for the CAT group (3.74 ± 0.34), while the lowest color change was recorded for the PK group (2.95 ± 0.45).

### 3.2. Flexural Strength (FS) and Probability of Survival Prediction

**Figure 2:**
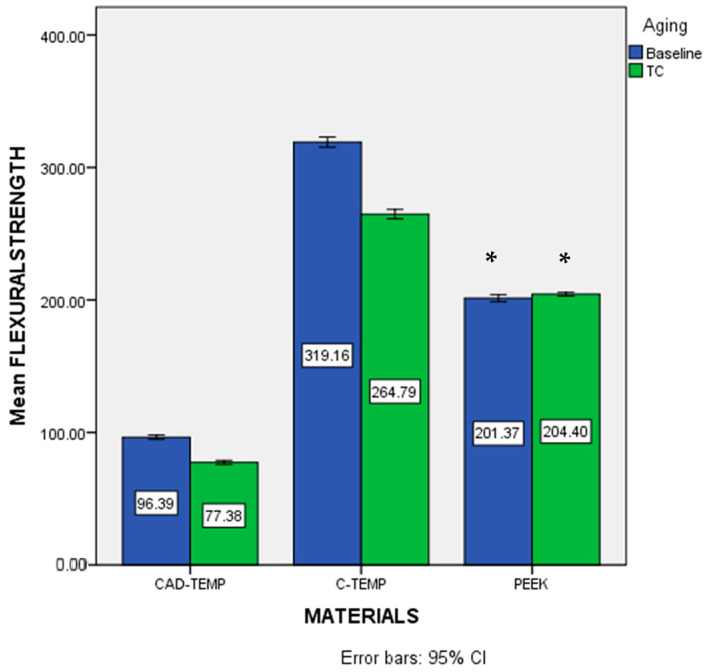
Flexural strength data for all groups (bars with the same asterisk indicate non-significant difference (*p* ≥ 0.05). Error bars represent a 95% Confidence Interval.

Table 4 shows the (means ± SD) of flexural strength values and survival probability for all groups. The type of material (*p* < 0.001) and thermocycling (*p* < 0.001) significantly affect FS and color stability values according to the two-way ANOVA statistics. Tukey’s multiple comparison tests revealed that the CT group had the highest flexural strength value among the baseline groups (Figure 2). The CAT group recorded the lowest flexural strength value. Thermocycling led to a significant reduction (*p* < 0.05) in flexural strength values for both the CAT and CT groups. On the other hand, there was no significant reduction in FS values for the PK group (*p* = 0.16).

Table 4 displays the Weibull parameters for all groups. The Weibull modulus values changed according to the material type and aging conditions. The Weibull modulus (m) and the characteristic flexural strength values (σ_0_) represent the cumulative survival probability. The Weibull modulus (m) for the CT group was significantly higher (*p* < 0.05) compared to other groups. Additionally, the characteristic strength for the CAT group was significantly lower than that of the other groups.

#### Scanning Electron Microscopy Evaluation

**Figure 3:**
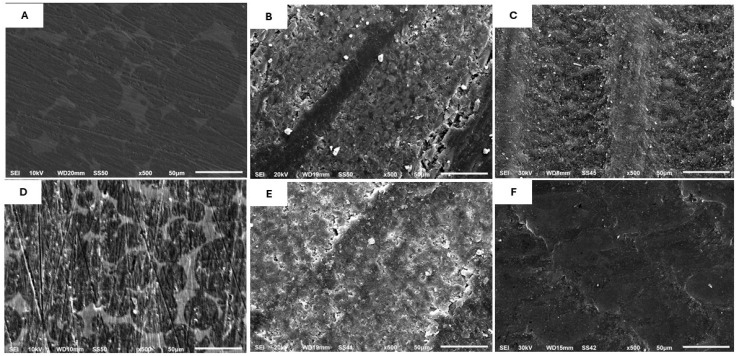
SEM micrographs (500×) of CAD-Temp (**A**,**D**), C-Temp (**B**,**E**), and PEEK (**C**,**F**) before and after thermocycling, respectively.

The SEM analysis showed variations in the surface microstructures of CAD-Temp, Everest C-Temp, and PEEK (Figure 3A–C). CAD-Temp displayed spherical areas of diverse sizes embedded within the resin matrix material (Figure 3A). The C-Temp exhibited irregularly elongated fibers interspersed among uniformly distributed spherical filler particles inside the materials (Figure 3B). The PEEK group exhibited a uniform surface texture without any voids or flaws (Figure 3C). CAD-Temp was the most affected surface, showing cracks after thermocycling (Figure 3D).

## 4. Discussion

Provisional restorations can be fabricated manually or with CAD/CAM technology. Manual fabrication presents various challenges, including unpleasant odors, considerable polymerization shrinkage, diminished durability, porosity, and increased surface roughness [29]. CAD/CAM provisional restoration is composite resin block that is produced from high-density preprocessed polymer, exhibiting greater edge stability that facilitates a better milling process with reduced thickness, improved polish ability, and enhanced mechanical properties [30,31].

Patients consistently express concerns regarding the aesthetics and functionality of their provisional restorations, particularly in cases where comprehensive occlusal reconstruction is necessary [32,33]. The clinical longevity of long-term provisional restorations is essential for their efficacy and success in oral settings. Various resin CAD/CAM materials are available in the market to fabricate provisional restorations. Their composition, microstructures, and oral environmental conditions affect their color stability and mechanical properties [13,14]. Due to the limited studies on the clinical performance of these materials, the current study aimed to evaluate the influence of aging on the color stability and mechanical properties of three different types of CAD/CAM polymer used for long-term interim restorative materials. This study demonstrated that CAD/CAM materials and thermal cycling had a significant impact on color measurement and flexural strength, as determined by the two-way ANOVA test. The null hypotheses were thus rejected.

Thermal cycling is a useful method for accelerating artificial aging and evaluating the clinical longevity of restorative materials by simulating the temperature variations in the patient’s mouth. Moreover, it has been reported to adversely affect restorative materials’ color and mechanical characteristics. The thermal aging protocol consisting of 5000 thermal cycles replicates a clinical duration of 6 months [15].

CIELAB evaluated the color difference between the tested materials. To determine the real clinical significance of the findings, the analysis of color difference must be associated with the perceptible (PT) or clinically acceptable (AT) thresholds for color difference. The thresholds for color differences that are perceptible or clinically acceptable depend on the references that are utilized [34,35]. Studies indicated that 50% of humans can detect color variations greater than one unit (ΔE > 1) [34,35]. There is a controversy about the acceptable color difference threshold. The acceptable threshold values that have been suggested by different studies range from ΔE* ≤ 2.35 to ≤3.7 [36,37,38,39,40]. The acceptable threshold ΔE* ≤ 3.3 was taken as a reference point in our study [39].

The color difference results were converted into NBS-ranging units for clinical comparison. The color difference in this study was (3.74 ± 0.39), (3.51 ± 0.54), and (2.95 ± 0.45) for CD, CT, and PK, respectively. Therefore, the color difference for this study was a marked color change, which is clinically unacceptable, except for that PK was perceivable but remained clinically acceptable. The findings of the current study align with those of Porojan et al. [41], who evaluated the color stability of PEEK concerning water saturation and thermocycling (10,000 cycles). They demonstrated that thermocycling did not affect the color of PEEK, with no marked color changes observed, only a slight color change.

The variations in the chemical composition of provisional restorative materials could explain the differences observed in color changes. It has been reported that the organic matrix mostly causes discoloration [34]. CAD-Temp has increased percentages of the organic matrix (83–86 wt.% PMMA) with lower filler particles, which is correlated to high-water sorption and lower color stability [4,42]. PEEK is a strong cross-linked polymer. It has been reported that PEEK showed the lowest water sorption compared to other PMMA-based and composite resin materials [19,20]. Although C-Temp is composed of a high-performance endless molecular polymer plastic chain, it recorded a marked change in color similar to CAD-Temp [21]. The presence of water may be responsible, as it can lead to corrosion of the glass fiber surface [43]. Furthermore, the findings of this study are consistent with those of Papathanasiou et al. [9], who indicated that aging procedures affect the color of CAD/CAM composite materials, which may compromise their aesthetic properties. It is essential to highlight that the aging procedures in this study included 10,000 thermocycles, while our study employed 5000 thermocycles. Accordingly, the first null hypothesis was rejected.

The resin matrix, along with the quantity and distribution of fillers, influences the flexural strength. At baseline, the C-Temp group exhibited the highest flexural strength in comparison to the other groups. This may be due to the exceptional performance, boundless molecular polymer resin matrix, and long glass fiber content [21]. This could be supported, as shown in Figure 3B by the presence of irregularly elongated fibers interspersed among uniformly distributed spherical filler particles inside the materials. Previous studies demonstrated that fiber-reinforced composites have significantly superior fracture toughness compared to traditional resin-based composites [43,44]. However, thermocycling significantly reduced the flexural strength in this group. This might be attributed to the corrosive impact of the aqueous environment on the glass fiber surface, leading to water infiltration through the polymer matrix and subsequently reducing the flexural strength [43]. These findings are consistent with another study [45], indicating that the flexural characteristics of CAD/CAM fiber-reinforced composite resin significantly reduced following one week of water immersion. This can be explained by the varying microstructure; CAD-Temp showed a notable polymeric content (83–86 wt.%), while PEEK is a cross-linked polymer with 20% ceramic filler, which can efficiently seal the gaps between the chains of the PEEK polymer. This was supported as shown in Figure 3A,C, where CAD-Temp displayed spherical regions of varying dimensions embedded within the resin matrix material, while PEEK exhibited a uniform surface texture without any voids. Conversely, the flexural strength of the PK group was not significantly impacted by thermal cycling. These results align with previous studies, reported no significant influence of storage conditions or thermal cycling on the flexural properties of PEEK [46,47]. Additionally, the water absorption rates for the PK group are (≤6.5 μg/mm^3^), for CAT (≤40 μg/mm^3^), whereas for the CT group, it is (9.6 μg/mm^3^) based on the manufacturer’s specifications [20,21]. Additionally, Juntavee et al. evaluated how aging affects the flexural strength of provisional materials utilized in oral rehabilitation. They utilized 5000 thermocycles and found that aging reduced the flexural strength of Vita CAD-Temp [48]. As a result, the second null hypothesis was rejected.

The validation of laboratory data for clinical purposes was supported by employing Weibull analysis, an effective approach for evaluating fracture behavior through the examination of data distribution instead of relying on mean values [12,26]. The Weibull modulus and characteristic strength are the statistical parameters commonly used to indicate the reliability of flexural strength data. Materials with higher Weibull modulus values are associated with increased reliability. C-Temp and PEEK recorded the highest Weibull modulus (48.02–50.29) and (43.46–45.48), respectively, compared to CAD-Temp. Considering the minimum clinically acceptable flexural strength value [25] (50 MPa), all baseline groups (86.39–303.94 MPa) and thermal cycled groups (70.06–251.51 MPa) had satisfactory strength values at a 95% probability of survival. Thus, the survival probability of C-Temp is higher than that of other tested materials.

Color stability and flexural strength may significantly impact the longevity of CAD/CAM provisional restorations, particularly when they are subjected to long-term clinical usage and environmental conditions. Furthermore, clinicians must possess the necessary knowledge to evaluate the latest available materials to identify the most appropriate options for long-term provisional restorative materials. This study has some limitations; only three provisional materials were utilized, so this investigation’s results cannot be generalized to other commercially available materials. The aging condition only simulates six months of clinical service. Additionally, other oral environmental factors need further investigation, such as saliva components, different levels of pH, and different staining solutions that could influence color stability and flexural strength. Further investigation should be performed considering a digital image system to simulate the clinical setting instead of utilizing one measuring technique (spectrophotometer).

## 5. Conclusions

Within the limitations of this study, the color measurement and flexural strength outcomes were significantly influenced by CAD/CAM materials and thermal cycling. The CT group demonstrated superior flexural strength compared to the other groups, both before and after thermal cycling. The PK group shows the lowest color change compared to other groups. Regardless of aging condition, C-Temp and PEEK materials recorded the highest survival probability, at a 95% significance level compared to CAD-Temp.

## Figures and Tables

**Table 1:** Materials utilized in the current study.

Product	Composition/Manufacturer	Indication	Lot. No.
CAD-Temp	-PMMA (83–86 wt.%), silica micro filler (14 wt.%) micro, Pigments (<0.1%). -VITA Zahnfabrik, Germany.	Long-term provisional restoration (multi-unit, fully or partially anatomical) up to two pontics.	38590
Everest C-Temp	-High performance polymer reinforced with glass fiber. -KaVo, Biberach, Germany.	Long-term temporary restoration up to 6 units.	6946
Bre CAM Bio HPP	-Poly ether ether ketone, 20 wt% titanium dioxide ceramic filler, and aluminum oxide sand (50 µm mean particle size). -Bredent GmbH, senden, Germany.	4-part posterior bridge up to two pontics.	56654456

**Table 2:** Level of color change according to NBS [22].

NBS Units	Color Changes
0.0–0.5	Extremely slight change
0.5–1.5	Slight change
1.5–3.0	Perceivable
3.0–6.0	Marked change
6.0–12.0	Extremely marked change
12.0 or more	Change to another color

**Table 3:** Color measurements for all groups.

Materials	Before Thermocycling			After Thermocycling				Color Changes
	L*	a*	b*	L*	a*	b*	ΔE*	ΔE* According to NBS
VT	65.94	−0.89	7.05	63.15	1.29	8.93	4.06	3.74 Marked change
	(±0.39)	(±0.04)	(±0.33)	(±0.49)	(±0.05)	(±0.15)	(±0.15)	(±0.39)
CT	66.93	−0.42	6.44	64.75	1.83	7.98	3.81	3.51 Marked change
	(±0.48)	(± 0.05)	(±0.15)	(±0.79)	(±0.03)	(±0.21)	(±0.18)	(±0.54)
PK	69.37	−1.04	9.23	67.23	0.53	10.73	3.21	2.91 Perceivable
	(±0.42)	(±0.04)	(±0.52)	(±0.32)	(±0.19)	(±0.34)	(±0.38)	(±0.45)

**Table 4:** Flexural strength and Weibull analysis for all groups.

CAD/CAM Materials	Flexural Strength	Weibull Parameters					
		m	r	σ_0_	FS at P_s_		
					5%	90%	95%
CAD-Temp Baseline	96.39 ^d^ (± 3.77)	25.68	0.85	96.95	101.18	88.82	86.36
CAD-Temp After TC	77.38 ^e^ (± 2.98)	27.64	0.93	78.04	81.22	71.92	70.06
C-Temp Baseline	319.16 ^a^ (± 6.92)	50.29	0.95	322.42	329.54	308.31	303.94
C-Temp After TC	264.79 ^b^ (± 5.91)	48.02	0.92	276.55	273.73	255.30	251.51
PEEK Baseline	201.37 ^c^ (± 5.00)	43.46	0.96	203.87	209.07	193.58	190.39
PEEK After TC	204.40 ^c^ (± 2.31)	45.48	0.92	205.58	207.98	200.74	199.22

m: Weibull modulus, r: correlation coefficient, σ_0_: characteristic flexural strength, FS: flexural strength; P_s_: survival probability. Mean values represented with different superscript lowercase letters is significantly different (*p* < 0.05).

## Data Availability

The original contributions presented in the study are included in the article, further inquiries can be directed to the corresponding author.
